# Value for money of medicine sampling and quality testing: evidence from Indonesia

**DOI:** 10.1136/bmjgh-2024-015402

**Published:** 2024-09-23

**Authors:** Sara Valente de Almeida, Katharina Hauck, Sarah Njenga, Yunita Nugrahani, Ayu Rahmawati, Rahmi Mawaddati, Stanley Saputra, Amalia Hasnida, Elizabeth Pisani, Yusi Anggriani, Adrian Gheorghe

**Affiliations:** 1 Department of Infectious Disease Epidemiology, Imperial College London School of Public Health, London, UK; 2 Nuffield Department of Population Health, University of Oxford, Oxford, UK; 3 Center for Pharmaceutical Policy, Management and Services Studies, Faculty of Pharmacy - Universitas Pancasila, Jakarta, Indonesia; 4 Erasmus School of Health Policy & Management, Erasmus University Rotterdam, Rotterdam, Netherlands; 5 Policy Institute, King's College London, London, UK

**Keywords:** Pharmacology, Public Health, Global Health, Cross-sectional survey

## Abstract

**Background:**

Substandard and falsified medicines (SFMs) are a public health concern of global importance. Postmarket surveillance in the form of medicine sampling and quality testing can prevent and detect SFM, however, there is remarkably scarce evidence about the cost and value for money of these activities: how much do they cost and how effective are they in detecting SFM?

**Methods:**

Between February and October 2022, Systematic Tracking of At Risk Medicines (STARmeds) collected and analysed for quality 1274 samples of 5 medicines from physical and online retail outlets in 7 Indonesian districts. We collated data on the resources consumed by STARmeds, related to all stages of medicines sampling and quality testing including design, fieldwork and laboratory analysis. We used activity-based costing principles to calculate the financial and economic cost of medicine quality surveillance from the perspective of a hypothetical medicines’ regulator. We calculated the cost per day and per week of fieldwork, per sample collected and per substandard sample. We used bootstrapping to capture uncertainty in the number of samples collected, by seller location type (urban, rural and online).

**Results:**

The total cost of sampling and testing medicines from the market was US$712 964 (current 2022 values). Laboratory costs represented the largest share (70%), followed by other direct costs (12%) and indirect costs (7%). On average, it costs STARmeds US$479 (95% CI US$462 to US$516) to collect one medicine sample and US$5990 (95% CI US$5601 to US$6258) to identify one substandard sample.

**Conclusion:**

Our findings bring urgently needed and novel information on the cost and value for money of medicine quality surveillance. These may support planning and budgeting of the Indonesian pharmaceutical regulator, but also of regulators and researchers elsewhere, particularly in low-income and middle-income settings, as well as international organisations with health regulation and quality of care remits.

**WHAT IS ALREADY KNOWN ON THIS TOPIC**

- Substandard and falsified medicines (SFMs) are a global concern as they threaten progress towards universal health coverage by wasting resources and potentially harming patients.
- Post-market surveillance of medicines is one of multiple strategies that can identify and potentially deter the production and distribution of SFM.
- Evidence is very scarce on the cost and value for money of medicine sampling and quality testing, particularly in constrained-resource settings.

**WHAT THIS STUDY ADDS**

- We present novel, detailed and transparent information on the financial and economic costs of studying medicine quality using primary data from a large study in Indonesia, including an exploration of alternative hypothetical scenarios.
- We found that the costs of laboratory testing of sampled medicines are the main determinant of cost and value for money of this type of medicine quality study.

**HOW THIS STUDY MIGHT AFFECT RESEARCH, PRACTICE OR POLICY**

- Researchers can use our findings to design, budget for, plan and report similar studies in other settings.
- Policy-makers can use our findings to articulate how to measure the value of studying medicine quality their setting, how to allocate appropriate resources for this and complementary strategies to ensure medicines quality.

## Introduction

 Substandard and falsified medicines (SFMs) are acknowledged as a public health concern of global importance.^1^ ^2^ ^3^ For countries striving to achieve universal health coverage, SFMs waste resources on products that are not fully serving their purpose and may even be harmful to patients.^4^ Following the definition from the WHO, substandard or ‘out of specification’, medicines are approved medicines that do not meet either the quality standards, their specifications or both while falsified medical products fraudulently misrepresent their identity, composition or source. National regulatory agencies are often responsible for deterring and identifying SFM production and dissemination, as well as following up on reported Adverse Drug Reactions.^5^ The general public’s increasing awareness and access to information promotes accountability and increases expectations towards the safety of medical products and services.^6^ As a result, cases of casualties from SFM have resulted in legal action against the government. This was recently the case in Indonesia, when tragically more than 200 children died after consuming cough syrup medicines.^7^ ^8^

While the prevalence of SFM is a global issue, regions with more cross-border movements, high demand for cheaper medicines and weak national regulatory policies appear more affected by this problem. In the Mekong subregion, for example, the prevalence of SFM among antimalarials is such that it increases drug resistance, which represents a step back in the progress towards malaria eradication.^9^ In a recently published systematic review of methods to measure the impact of SFM, authors found that substandard and falsified antimalarial medicines can account from 10% to 40% of total annual malaria costs, and SFMs affect rural and poor populations disproportionately. Further research is needed to understand the real magnitude of this issue since most studies on the impact of SFM were conducted in sub-Saharan Africa and focused on antimalarials and antibiotics only.^10^

Postmarket surveillance is a catch-all term for systems that monitor the safety of medicines already approved for use in a market. These include passive pharmacovigillance systems that rely on reports of adverse reactions, and active sampling and testing of medicines to check that products samples at different points in the supply chain continue to conform with their authorisation paperwork. National regulators may adopt either or both of these strategies. Ideally, the data from the two systems are cross-referenced to maximise the chance of detecting poor-quality medicines and removing them from the supply chain, although in practice this is not always the case.^11^ What postmarket surveillance entails in practice depends on the strategy adopted by national regulators, for example, health facilities reporting issues with a medicine (adverse events or evident signs of damage) to manufacturers and regulators after a product has been approved, conducting medicines sampling and quality testing activities from the supply chain. These activities may detect drug risks and potentially deter SFM production and dissemination in the first place.^12^ Fortunately, governments are taking action and medicines quality monitoring is an important part of future strategies to promote drug safety in Asia.^13^ Postmarket surveillance in the form of medicine sampling can prevent and detect SFM, however, there is remarkably scarce evidence about the cost and value for money of these activities: how much does it cost and how effective is it in detecting SFM? What are the government budget implications of sampling medicines from the market for quality testing?

The project Systematic Tracking of At Risk Medicines (STARmeds, https://starmeds.id) implemented in four areas of Indonesia (North Sumatera, Greater Jakarta, East Java and East Nusa Tenggara (NTT)) developed a single cross-sectional survey that can add information to the national medicine regulator’s model through collecting and testing medicine samples from urban and rural areas and all types of outlets from which patients acquire medicines.^14^ STARmeds collected samples of five medicines (amlodipine, amoxicillin, cefixime, dexamethasone and allopurinol) from a random selection of physical and online outlets. We defined a sample as a single medicine, dose and brand acquired at a single outlet and time. Taking a patient exposure rather than a regulatory approach, the study did not distinguish between different batches of a brand, as long as they were provided to a single patient at a single time and place.^15^ The collected samples underwent laboratory quality testing, as described below. A sample was considered substandard if it failed at least one of the chemical tests to which it was subjected. For the purpose of this analysis, we focus on the costs of finding substandard samples, which do not include cases of falsified medicines.

Using the results from STARmeds activities and laboratory testing as a benchmark, in this study, we estimated the costs of sampling medicines from the market and testing for medicine quality from the perspective of a medicines regulator, by adapting costs incurred in our academic research to reflect the different costs incurred when similar activities are implemented by a medicine regulator. With a combination of administrative records and time-and-motion survey tools, we aimed to:

1. Quantify the financial and economic costs of conducting postmarket surveillance based on active sampling of medicines from randomly selected dispensing points.
2. Calculate the cost per medicine sample collected and the cost per substandard sample found.
3. Provide practical and concrete guidance on the costs, resource use, budget impact and potential value of this type of medicine quality study.

We present findings from the STARmeds project that improve the available knowledge on resource requirements for sampling and testing medicines from the market, disaggregated by phase and cost centres, and hypothetical scenarios that can inform the budgeting and planning of similar activities.

## Methods

We follow the structure of the Global Costing Reference Case (GCRC) developed by the Global Health Cost Consortium. In online supplemental file 1, we show in detail how and where each principle in the GCRC is reported in our paper.

### Project STARmeds

STARmeds is an intersectoral study that joined together professionals from different backgrounds and institutions (Universitas Pancasila, Imperial College London and Erasmus University) to assess the prevalence of SFM in Indonesia. The core of STARmeds project was to conduct a cross-sectional survey of the quality of medicines at the point of dispensing to patients that contributed to identify and measure the magnitude of SFM prevalence in the country, and how likely are local patients to find SFM. The full scope of the project, that goes beyond testing medicine samples quality, can be summarised in five main objectives:

1. Implement medicine quality study activities—by collecting medicine samples in the field.
2. Estimate SFM prevalence—by testing samples collected in the lab.
3. Compute costs of implementing sampling and quality testing of medicines.
4. Understand policy influence and learning during the implementation of the project.
5. Inform policy research in Indonesia.

The objectives were divided into three work streams: one to implement a medicine quality study and produce substandard prevalence estimates, one to develop the costing analysis, and a policy learning module to study the role of policy engagement in medicine surveillance. In this study, we focus on the costing analysis work stream, dedicated to measure total costs, cost per sample collected and per substandard sample found. Calculations of costs per sample and per substandard sample found are built on the substandard prevalence estimates, which shall be published separately, together with a detailed description of the data collection and testing methods.^16^ ^15^

Between March and May 2022, STARmeds collected 1333 medicine samples of amlodipine, amoxicillin, cefixime, dexamethasone and allopurinol (see sample distribution per type of medicine in online supplemental file 2). In STARmeds, one sample means one unit of analysis in a medicine quality study. It comprises one medicine (eg, amoxicillin), of one dosage (strength and formulation) (eg, 500 mg tablets), of one brand (eg, Supermoxy), from one market authorisation holder (eg, Modern Pharma) collected at one location, at one time, (eg, bought at Rainbow Pharmacy, on 20 June 2022).^15^ Medicines were selected during the project’s preparation stage in consultation with the study’s Scientific Advisory Group members and representatives of the Indonesian food and drug regulatory agency Badan Pengawas Obat dan Makanan. Medicines were sampled from a random selection of outlets where patients can obtain medicines, either physically, in rural or urban settings, or online, across seven districts of four Indonesian areas: North Sumatera, Greater Jakarta, East Java, East Nusa Tenggara (NTT). Sampling locations were chosen purposively to reflect Indonesia’s geographical and economic diversity. 1274 were collected and sent for laboratory analysis for quality testing (identification, quantification and uniformity of assay, uniformity of content for samples below 20 mg, and dissolution). Of the total samples tested, 105 were found to be substandard, leading to a crude substandard prevalence of about 8.2% that shall be the value adopted for the costing analysis throughout this study. In the costing analysis, we do not include costs of finding falsified medicines, or of adjusting crude estimates of prevalence by market size. Methods on medicine quality testing followed MEDQUARG guidelines and details are published together with substandard prevalence estimates.^16^ ^17^

### Approach to economic analysis of STARmeds

Based on the sample design and prevalence estimates achieved with the medicine quality study, we conducted a microcosting study to inform policy decision-makers on the cost of sampling and quality testing of medicines in the market and its performance in finding substandard medicines in the market. Microcosting is a cost estimation method that uses detailed resource utilisation and unit cost data to generate precise estimates of economic costs.^15^ Costs, cost items and study phases were retrieved from the STARmeds project registry and from consultation with experts whenever STARmeds costs were not applicable to our adopted perspective of a hypothetical medicine regulator (the case of salaries, eg). We categorised STARmeds activities in three stages:

- Study setup (design and sampling frame) and preparation of sample collection (protocol, tools, stakeholder engagement, recruit data collectors, logistics and administration).
- Fieldwork (training sample collectors, travel, collecting samples, processing collected samples for laboratory analysis—including checking for anomalies in packaging or labelling).
- Laboratory analyses and reporting (testing, registering results and report writing for dissemination with relevant stakeholders).

For each stage, we identified activities and the required resources for implementing the medicines survey. We then assigned unit costs to resource items and calculated the respective total costs. We distinguish between financial costs, which include the costs of resources reflected in STARmeds’ expenditure records and economic costs, which also include the value of time, goods and services that were used in STARmeds’ activities but without a financial transaction.

### Calculating total and unit costs of medicine sampling and quality testing

We calculated full (not incremental, ie, assuming there was no other surveillance activity in place), real-world (not normative), financial and economic costs from the perspective of a hypothetical public sector medicines regulator (excluding research costs) using STARmeds activities as a benchmark. The time horizon covers the calendar period October 2021–October 2022, during which the STARmeds study design, data collection and laboratory analysis took place. The scope of the costing exercise includes all resources consumed by STARmeds related to all study stages. We did not include any research-related costs (such as literature reviews and scientific papers discussion, writing and publishing process), nor any potential administrative follow-up based on laboratory results, for example, communication with, investigation or inspection of manufacturers of substandard samples.

We used top-down costing as the main analytical approach. In addition to STARmeds financial and administrative records, we quantified and allocated STARmeds staff human resource costs based on civil servant’s salaries lists (including allowances) in consultation with our expert staff in the field.^18^ This consultation was part of a time-and-motion study that provided crucial information on overtime worked by staff. We included these as economic costs by multiplying their full-time equivalent (FTE) salaries by the estimated number of hours worked outside normal working hours (assumed to be from 9:00 to 17:00 hours). FTE here is defined as the hours worked divided by the total amount of hours in a full-time workweek (a full-time employee has an FTE of 1). Costs were computed assuming a hypothetical core team that remained stable and worked full-time throughout the whole project. The team, composed of a senior investigator, a logistics manager, a data manager and two research staff, was defined after consultation with STARmeds experts that work closely with medicine regulatory institutions. Table 1 summarises the data sources and assumptions made for each type of cost input when specifying the cost perspective of a hypothetical medicines regulator.

**Table 1:** Data sources and estimation methods for cost inputs

Cost input	Details
Staff salaries	Salary data for each team member were informed by the Indonesia civil service scale (see details in online supplemental file 4). In our hypothetical team, grade I civil servant corresponds to assistant level, grade II to data manager, grade III to project manager and grade IV to principal investigator. Each level receives an allowance (that increases with level of seniority) to cover extra benefits such as sick, maternity and annual leave, insurance, service car, etc. Medicine sample collectors are paid on a per diem basis, following STARmeds expenses records. Salaries and per diems, as appropriate, were allocated to the three study phases (study design, fieldwork and analysis) proportionally with the number of calendar days spent in each, based on STARmeds fieldwork operations. For the calculation of economic costs, staff overtime was valued at the same hourly rate as their full-time employed time.
Equipment	The cost of newly purchased equipment was informed by STARmeds expenditure records. When personal laptops and phones were used by the core team, purchase values of US$1000 and US$100, respectively, were used to compute economic costs, informed by prices of similar specifications to those used in STARmeds available on the e-catalogue website of Indonesia’s National Public Procurement Agency LKPP.^29 30^ In all cases, a useful life of 5 years and a discount rate of 3% were assumed to annualise equipment cost.
Consumables	All costs of consumables were informed by STARmeds expenditure records. COVID-19-related consumables were excluded, for example, rapid diagnostic tests.
Travel	This category includes travel, meals, accommodation and meeting-related costs, for example, training data collectors. In each geographical location from where samples were collected, there was a central coordination hub for which rent, utilities, buildings insurance were charged at market prices. Transport between Jakarta and the provinces, as well as local transport, are also included. All costs are informed by STARmeds expenditure records.
Laboratory	This category includes the costs of privately contracted Equilab to perform pharmacopeial tests (identification, quantification and uniformity of assay, uniformity of content for samples below 20 mg and dissolution) for 1274 medicine samples. These costs were registered as a whole item, as they were registered in STARmeds expeniture records.
Other direct costs	This category includes operational allowances for sample collectors, the cost of purchasing medicines from outlets at market prices, as well as a wide range of services, for example, barcoding samples, mobile data packages for the field team. All costs are informed by STARmeds expenditure records.
Indirect costs	It was assumed that overheads represent 10% of the sum of salaries, consumables, travel and other direct costs. This assumption results from consultations with STARmeds experts, based on the STARmeds project and Indonesian civil service earnings.

STARmeds, Systematic Tracking of At Risk Medicines.

The sampling frame for the cost analysis is the same as the sampling frame of the STARmeds project. STARmeds collected and tested 1274 samples. Of these, 327 samples were collected online (sample distribution between medicines in online supplemental file 2). STARmeds Guide and Toolkit include a version of the study design description adaptable to many settings in chapter 3 (see online supplemental file 3) and the units of analysis for the costing study are defined based on information in STARmeds administrative records.^14^

In the cost analysis results, the term ‘cost per medicine sample collected’ refers to the cost of a sample collected during fieldwork (as per the definition stated in the ‘Methods’ section) which underwent visual inspection. When we report the ‘cost per substandard sample’, we mean a collected sample that underwent visual inspection, was sent to the laboratory for analysis and failed at least one laboratory test in any of the chemical tests performed: identification, quantification and uniformity of assay (percentage of labelled amount of active pharmaceutical ingredient (API) present in sample), uniformity of content for samples below 20 mg (distribution of the active content within the production batch) and dissolution (percentage of labelled amount of API dissolved within specified time frame).^15^ ^14^

All costs were incurred in Indonesia in calendar year 2022. Capital expenditure comprised only computers and mobile devices to support fieldwork. Capital was depreciated using the straight-line method, assuming 5 years of useful life and a 3% discount rate.

All costs are reported in US dollar (USD, current 2022 values). Since STARmeds expenditure reports were submitted in GBP to the study funder, these amounts were converted to USD based on the average Pound Sterling (GBP) to USD exchange rate in 2022. Other costs not incurred in GBP were converted from Indonesian Rupiahs (IDR) to USD using the average IDR to USD exchange rate for 2022. Given the 1-year horizon (October 2021–October 2022), no discounting was applied.^19^ ^20^

We complemented the costing analysis with information from a time-and-motion study to account for overtime in our salaries’ estimates. STARmeds staff involved in data collection activities recorded time taken for different tasks during fieldwork, and filled in individual time-use questionnaires. In addition, we held three focus group discussions with data collection staff approximately 6 months after the last medicine sample was collected in fieldwork, to discuss time use.

Apart from overtime, the time-and-motion study collected information that is only relevant for planning purposes and that was not directly included in the cost estimations. The methods used for the time-and-motion study are described in detail in online supplemental file 5.

### Sample collection estimates and uncertainty

Our estimates of total costs and unit costs account for uncertainty in the number of samples collected per day, which influences the number of fieldwork days during which the target number of medicine samples can be collected and, in consequence, the total costs of keeping the team (including sample collectors) in the field. We did so by bootstrapping (sampling with replacement, n=500 iterations) the number of samples collected during each day of fieldwork, stratified by type of outlet location (rural, urban and online). For each iteration, we calculated the number of days required to reach the target number of medicine samples to be collected and then, across all iterations, we calculated the mean and the 95% CI (percentile method). In this context, and from the information collected during the focus groups, we considered potential sources of uncertainty in the number of samples collected per day the weather conditions, stock shortages or other unexpected events such as road accidents or health issues.

### Sensitivity analysis

To understand how our results would change if some key expenses varied, we conducted a one-way sensitivity analysis, with three alternative scenarios, where we modified the (A) costs of sampled medicines, (B) sampling and testing preparations costs and (C) substandard prevalence estimates, alternately (keeping all other costs constant). In scenario A, we multiplied prices of medicines in STARmeds by 10, to simulate the survey costs if more expensive medicines had been sampled. In scenario B, preparation costs (including costs with study design, operational preparations, stakeholder engagement and recruitment of data collectors) were halved, to account for the fact that this stage is likely to become less resource-intensive with time and repetition. Finally, because prevalence estimates can vary with medicine types, in scenario C, prevalence was increased to 20%, instead of the 8.2% found in STARmeds laboratory testing (assuming the same cost of laboratory testing).

### Patient and public involvement

There was no patient involvement in the development of this project.

## Results

**Figure 1:**
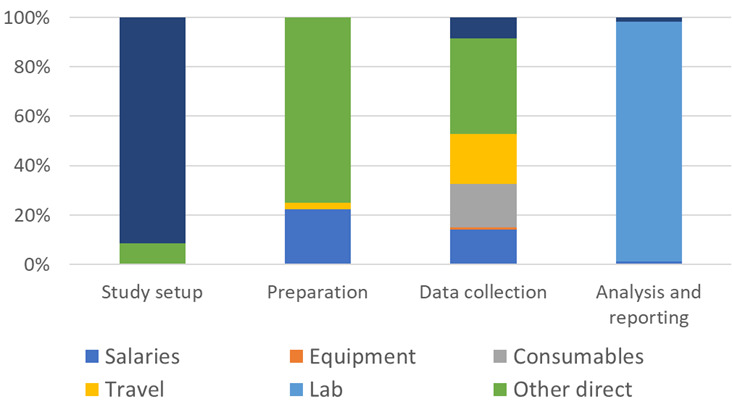
Breakdown of cost components by study phase in STARmeds project. Study setup: 1 October 2020–30 September 2021; Preparation: 1 October 2021–12 February 2022; Data collection: 13 February 2022–9 May 2022; Analysis and reporting: 10 May 2022– 30 June 2022. Study setup and preparation of sample collection include overall design, sampling frame, protocol, tools, logistics and administration; fieldwork section includes training sample collectors, travel, collecting samples, processing collected samples for laboratory analysis: and analysis and reporting includes laboratory testing and results.

The total cost of medicines sampling and quality testing (under STARmeds) was US$712 964 (table 2). Laboratory costs represented the largest share (70%), followed by other direct costs (12%) and indirect costs (7%). Consequently, ‘analysis and reporting’ was the most financial resource-intensive phase; by comparison, data collection accounted for about 15% of total costs. ‘Other direct costs’ (that includes costs of buying samples) accounted for the largest share of costs in preparation and data collection while ‘laboratory costs’ dominated ‘analysis and reporting’ (figure 1).

**Table 2:** Disaggregation of total costs and unit costs of STARmeds activities (USD, current 2022 values)

		% of total cost
By cost components		
Salaries*	US$ 32 503	5
Equipment	US$ 2975	0.4
Consumables	US$ 17 747	3
Travel	US$ 21 660	3
Laboratory	US$ 481 704	70
Other direct	US$ 82 734	12
Indirect	US$ 51 200	7
Total	US$ 690 523	100
By study phase		
Study setup	US$ 37 296	5
Preparation	US$ 54 356	8
Data collection	US$ 100 508	15
Analysis and reporting	US$ 498 363	72
Total	US$ 690 523	100

	Units	Avg.	(95% CI)
Samples collected	1274	US$ 479	(462; 516)
Substandard detected	105	US$ 5 812	(5601; 6258)
Fieldwork day (all)	61	US$ 1 866	(1482; 2728)
Fieldwork day (active)	49	US$ 2 293	(1821; 3353)

Components and study phases are taken from STARmeds project administrative records. STARmeds conducted medicines postmarket surveillance activities in four medicines in Indonesia. These activities correspond to the costs associated to preparing, collecting and testing 1274 medicines in physical and online outlets from February to May 2022. Fieldwork day (all) corresponds to the cost per calendar day of the field work period, fieldwork day (active) corresponds to cost per days when at least one sample was collected, both excluding laboratory costs.

* Salary scale list presented in online supplemental file 4, online supplemental table 4A.1.

STARmeds, Systematic Tracking of At Risk Medicines.

The mean cost of collecting samples was US$479 (95% CI US$462 to US$516) to collect one medicine sample and US$5812 (95% CI US$5601 to US$6258) to identify one substandard sample (ie, collect, test and report), assuming a prevalence of 8.2% substandard in the market (table 2). This prevalence estimate falls to 4.4% when adjusted for market size, meaning an increase in the average cost per substandard sample to US$ 10 883 (95% CI US$10 482 to US$11 888).^15^ Laboratory testing alone cost on average US$378 per sample, which means around US$100 was spent on design, fieldwork and reporting. In terms of time, each fieldwork day costs US$1866 and each fieldwork week US$9330 (assuming a working week of 5 days)—excluding laboratory costs, as they were accrued afterwards.

Table 3 outlines the number of overtime hours worked during STARmeds activities, based on data from individual questionnaires and focus groups with STARmeds research staff. Data collection was the most demanding project phase in terms of workload since it required staff to work overtime almost every working day. The amount of overtime was equivalent to four conventional working weeks (5 days working from 9:00 to 17:00 hours) for the preparation of data collection, 6 weeks for fieldwork and 3 weeks for stakeholder engagement.

**Table 3:** Operational efficiency indicators for STARmeds project

Unit	Preparation	Data collection	Stakeholder engagement
Total hours worked per week (hour)	15.0	30.5	5.3
Weeks executed (weeks)	25.8	12.9	34.4
Weeks needed to avoid overtime (weeks)	29.4	18.5	37.3

Data from individual questionnaire and focus groups. Assuming total hours worked includes overtime, we calculate the number of hours worked without overtime; Based on focus groups discussion extra working time in a day was assumed to be 3 hours for preparation, 5 hours for data collection and 2 hours for stakeholder engagement.

STARmeds, Systematic Tracking of At Risk Medicines.

### Sensitivity analysis

**Figure 2:**
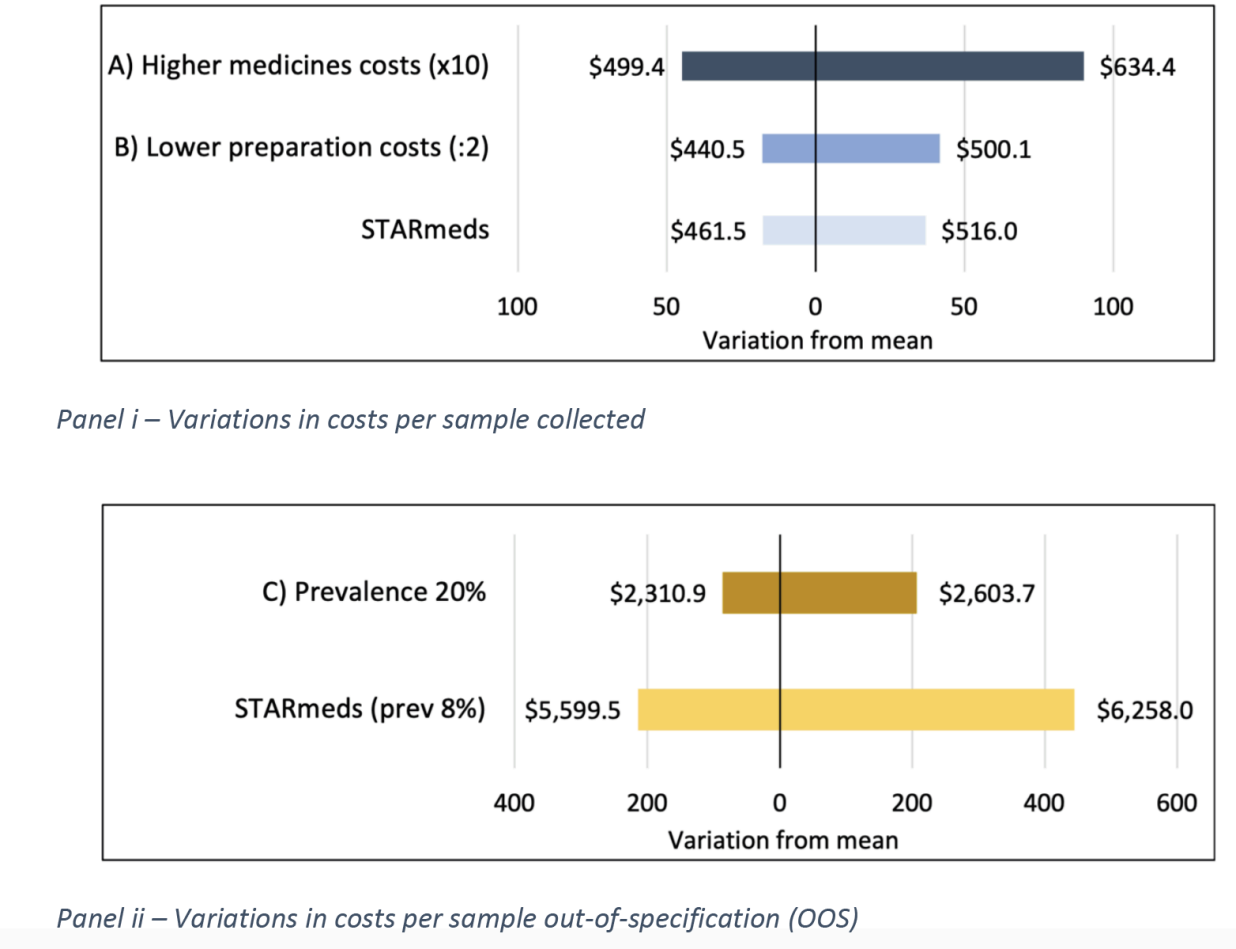
Scenario analyses for unit cost estimates of STARmeds project. STARmeds values correspond to the average cost per collected sample estimated using STARmeds data and activities. In panel (i), we compare these estimates to other hypothesis and study how costs would vary in case (A) the medicines sampled were 10 times more expensive than STARmeds or (B) if preparation costs were half of that of STARmeds. In panel (ii), we estimate how costs would vary in case (C) the prevalence of medicines substandard is 20%, instead of the 8% found in STARmeds. STARmeds, Systematic Tracking of At Risk Medicines.

Results from sensitivity analysis show that because laboratory costs are the most relevant driver of total costs, changes to other key factors do not produce a large effect on the overall cost per unit. Figure 2, panel i (scenarios A and B), shows that spending 10 times more on medicines and half the observed costs on preparation for data collection would have produced similar results for the mean cost per sample collected and analysed.

For scenario C, increasing prevalence estimates from 8.2% to 20% had a significant and negative impact on cost per substandard sample found, which illustrates how relative costs increase when substandard medicines are more scarce and more difficult to find. From figure 2, panel ii, if prevalence of medicines substandard had been 20% the mean cost per substandard sample would have ranged from US$2311 to US$2604, about half the costs observed in STARmeds.

## Discussion

### Summary of findings

We calculated the total and unit costs of medicines sampling and quality testing, including the preparation of sample collection, sample collection, laboratory analysis and reporting, based on administrative records and primary workload collected as part of the STARmeds project activities in Indonesia in 2021 and 2022. The mean unit costs were US$479 and US$5812 per sample collected and per substandard sample detected, respectively. Results were not substantially affected by different assumptions about the market prices of sampled medicines and by more efficient sampling preparation.

Laboratory analysis for collected samples was the main driver of STARmeds costs, representing more than two-thirds of costs. Data collection was the most intense phase of the project and required staff to work outside working hours almost every day.

Our methods and findings are aligned with other studies on the costs of medical surveillance programmes (although not focused on medicine quality). A costing analysis of a surveillance programme for miners exposed to high altitude in Chile measured the direct operating costs of surveillance through on-site surveys and microcosting methods, quantifying labour costs, consumables, equipment and overhead costs.^21^ Similarly to our approach, authors use a time activity-based costing for measuring salary costs, although not accounting for staff overtime. The estimated average cost per worker was about US$21, and total programme costs were mostly driven by staff costs and high-cost equipment. In the Mekong subregion, a costing study was developed to document the costs of electronic systems and mobile reporting solutions as part of a Malaria surveillance programme.^22^ Authors include economic and financial costs to measure the annual expenses per provider under surveillance. The annual cost per provider varied from US$82 in Myanmar to US$371 in Cambodia, and US$354 in Laos, considered sustainable as it corresponded to 0.5%–1.5% of national malaria strategic plan cost and 7%–21% of surveillance budgets. In the sub-Saharan region, a review of the costs of home-based testing for HIV surveillance estimated an average cost of US$23 per person tested and US$439 per HIV-positive test. Studies in this review used either an ingredients-based costing approach or microcosting and all included staff salaries, per diems, transport, accommodation and equipment costs, with no mention of staff overtime.^23^ Just like there is scarce evidence on the costs of finding SFM, not much is known about the costs of testing and tracing COVID-19 cases in low-income and middle-income countries.^24^ In many countries, the pandemic placed an increased burden on regions that were already low on resources. A microcosting analysis from Ethiopia estimated that the total cost of COVID-19 laboratory diagnosis, including sample collection, was US$5.26 per test and US$49.33 per COVID-19 positive identified. As in the case of STARmeds, more than 70% of the total cost was spent on laboratory testing and the rest on data collection.

### Limitations

We have excluded research costs from our estimates and only included costs that would apply to a hypothetical national regulator. We acknowledge, however, that this is an approximation and we do not claim to have captured completely the operational costs of medicine surveillance activities that would be implemented by such a regulator, and since no regulatory authority was directly involved in the study. We have attempted to present our assumptions transparently to inform the contextualisation of our findings with due adjustment. Also, we did not factor in the cost of policy action towards substandard samples (eg, inquiries, checks, inspections, other administrative or legal action) or the potential deterrent effect of such action.

Focus group discussions with STARmeds staff were conducted retrospectively, 6 months after the fieldwork was completed, which raises the potential for recall bias when estimating the workload amount. Besides individual questionnaires on staff workload, we also collected time-stamp data recorded electronically and on paper by sample collectors. The latter data informed our understanding of the field workload, but not our main calculations as it had a lot of variation which may be due to different time perceptions across individuals and because recording timestamps in the field increased the burden on them.

The number and type of medicines collected by STARmeds are broadly used by many of us and constitute some of the cheapest medicines in the whole market. While our sensitivity analysis shows that variations in the price of medicines have a limited impact on the overall cost per sample, we acknowledge that the prices STARmeds paid for the medicine samples collected were low and may represent a bias in our estimates. In the same way, there are large ranges of laboratory testing costs between laboratories and type of molecule being tested, the key drivers of our total cost. Both these factors may affect generalisability of our results, which we try to (at least partially) overcome in our sensitivity and scenarios analyses.

### Interpretation and discussion of findings

Our findings suggest that sampling and testing medicines for quality are rather costly activities principally because of laboratory costs. We could not identify other similar studies on the surveillance of medicine quality to directly compare our findings with. While some characteristics of STARmeds contributed to high costs with a view to ensuring the findings’ representativeness and robustness, such as sampling from urban and rural locations as well as from a wide range of regulated and unregulated medicine sellers (including online), other characteristics favoured efficiency, for example, using experienced research staff with a pharmacy background and relying on sample collectors with extensive survey experience on the National Statistics Office roster. Even if a hypothetical regulator may approach and conduct medicines surveillance activities differently in the same context, potentially generating economies of scale and scope in selected activities, the fact that laboratory costs are the major driver of total costs makes it unlikely that total and unit costs would have been meaningfully different if the same sampling design had been implemented differently.

Despite salary costs representing less than 10% of total costs, the workload required to conduct the fieldwork activities was particularly intense. This was even more the case for online medicine sellers: instances such as fluctuating responsiveness and last-minute delivery cancellations kept STARmeds research staff occupied for as long as several hours to have a viable medicine sample purchased and delivered. For all types of activities, research staff had to work overtime, especially during the data collection period. The results of the time-and-motion component showed that considering only financial, objective costs from administrative records would fail to acknowledge human factors that are crucial for the successful planning and implementation of medicine surveillance activities.

### Implications for policy-makers

Ascertaining the value of postmarket surveillance activities requires policy-makers deciding on its objectives and resources to consider carefully a range of contextual aspects. An important initial distinction is between one-off medicine quality exercises and routine postmarket surveillance. While our cost estimates are based on STARmeds, a standalone study, having these cost estimates inform a discussion on the value of postmarket surveillance in the form of medicine sampling first requires being explicit about what are the policy objectives, what type of study design best serves these objectives (eg, random or targeted sampling) and what ‘value’ represents in context.

If ‘value’ refers to the amount and quality of existing knowledge on the substandard medicines prevalence in the respective context, then it can be argued that with absent credible information on the likely extent of the problem, for example, a STARmeds-like type of exercise can offer novel, otherwise unavailable insights that can focus the policy dialogue and serve as a benchmark for future medicine quality efforts. With ‘value’ understood as knowing the likelihood of a patient buying substandard products, then the substandard point prevalence is determinant since the average cost of detecting one substandard sample was largely determined by the prevalence and laboratory testing costs, as illustrated in our scenario analyses (figure 2). A recent study on the return on investment of technologies to detect substandard and falsified amoxicillin focuses on this issue, and studies the possibility of using more cost-efficient screening alternatives to quality testing, leaving standard quality tests for measuring APIs (such as the high-performance liquid chromatography technology used by STARmeds) for confirmatory tests.^25^ As illustrated in scenario C (panel ii), randomised Post-market surveillance (PMS) has diminishing returns to prevalence: the smaller the number of substandard medicines in the market the more expensive and the more difficult it is to find them. One can argue that preventing the existence of substandard medications in the market is a balance between investing in Good Manufacturing Practice (GMP) inspection and enforcement, and PMS since effective quality control at production can reduce the need for extensive (and expensive) randomised PMS. In that sense, when there are not enough substandard medicines in the market to justify the cost of randomised PMS, risk-based surveillance might be a better option, which requires a different cost-effectiveness study. On the other hand, countries with a significant number of substandard products should prioritise random PMS. How many substandard medicines constitute a ‘significant’ amount to justify this view of ‘value’ of PMS is a trade-off that national regulators need to take into consideration in their planning activities. When ‘value’ is understood as preventing or deterring substandard products from being brought to the market, the higher the magnitude of this deterrent effect, the higher the value of the postmarket surveillance activity and the lower the expected prevalence of SFM in the market. While, to our knowledge, there is no empirical quantitative evidence on the deterrent effect of medicine surveillance activities, criminological research has found significant deterrent effects associated with the use of surveillance cameras (with random or targeted installation), by increasing the criminals’ expected probability of being detected.^26^ ^27^ ^28^ If postmarket surveillance activities are known to the people and work as surveillance cameras for the medicines market, criminals may be discouraged to produce or sell SFM, just by the existence of these activities. An information experiment increasing the visibility and awareness of surveillance activities in the form of medicine sampling and testing from the market could be useful to investigate the existence and pontetial of this effect. The same would apply if ‘value’ refers to avoiding adverse events due to SFM: the more cases are avoided, the higher would be the value of postmarket surveillance. Studies estimating the economic impact of SFM have included costs related to adverse events, which could help understand the value of avoiding them, but this evidence is limited to sub-Saharan Africa countries and antimalarials or antibiotics.^10^

Another aspect worth considering when discussing the value of studying medicine quality is that postmarket surveillance by itself cannot be expected to successfully address the SFM problem; it is one strategy among many that should be viewed as part of multifaceted approach, in line with the available conceptual frameworks. In that respect, the value of postmarket surveillance by medicine sampling and testing can only be ascertained in combination with the other policy strategies that are in place in the respective setting, from inspections at production level to securing safe supply chain for medicines or promoting innovation in medicines technology.

Finally, costs and value estimations are not to be read as a justification for decreasing postmarket surveillance activities, especially in low or constrained resource settings. Rather they bring more information on how to plan these activities in a way that maximises its sustainability and efficiency. With our estimates, we hope to contribute valuable information to PMS budget planning estimations that can help researchers and regulators make informed decisions on the trade-offs that these activities require to ultimately find efficient and sustainable ways of ensuring universal access to quality medicines and reduce the prevelance of substandard and falsified products as much as possible.

### Implications for research

While other studies in the literature have analysed costs of different types of surveillance,^21^ ^22^ ^24^ ^25^ to our knowledge, ours is the first study that provides a comprehensive, detailed and transparent breakdown of the costs of medicines sampling and quality testing. Our expectation is that our approach and results will be useful, with adaptations, to researchers and regulatory authorities planning and budgeting for the future of such studies in other settings.

To support such applications, we have developed based on our STARmeds work a free, web-based tool to support researchers and regulators in the ex ante budget planning of medicines quality surveillance activities, available at https://shiny.dide.ic.ac.uk/starmeds-budget-tool/.

Recommendations for future research on the value of medicines quality surveillance include that there is no empirical, quantitative evidence on the subject and more research is needed to establish the extent to which a deterrent effect is plausible (other than in theory) and, if so, what could be its range. Beyond postmarket surveillance, there is a dearth of economic evidence on the costs of other strategies for SFM prevention, detection and action, for example, GMP inspections. Future studies can fill this gap and ultimately produce comparative value estimates among complex SFM strategies with multiple, integrated components, not only isolated interventions such as medicine quality surveillance.

## Conclusion

Our findings suggest that surveillance of medicine quality at randomly selected dispensing points is a costly activity whose value is highly contextual and difficult to quantify given the available empirical data. Regulators should adapt their methods to match their goal when developing medicine surveillance activities in the supply chain. We provide novel information that may prove useful to researchers and regulators finding this match, particularly in constrained resource settings, but also to international organisations with a healthcare quality remit. Building information and creating tools for planning and budgeting of medicine quality studies is essential for improving resource use and designing efficient evidence-based policies to tackle the prevalence of SFM.

## Supplementary material

10.1136/bmjgh-2024-015402 online supplemental file 1

10.1136/bmjgh-2024-015402 online supplemental file 2

## Data Availability

Data are available on reasonable request.
