# Supplementary material for: Value for money of medicine sampling and quality testing: evidence from Indonesia
Source: BMJ Glob Health. 2024 Sep 23;9(9):e015402. doi: 10.1136/bmjgh-2024-015402 (PMC11429347; doi:10.1136/bmjgh-2024-015402)
Supplement: online supplemental file 1 [file bmjgh-9-9-s001.pdf]

Supplementary File 1

A. Acronyms list

ADR	Adverse drug reaction
BPOM	Badan Pengawas Obat dan Makanan
FTE	Full time equivalent
GBP	Pound Sterling
GCRC	Global Costing Reference Case
IDR	Indonesian Rupiah
NTT	East Nusa Tenggara
OOS	Out-of-specification (medicines that failed in at least one of the quality tests performed and belong to the category of substandard medicines)
SFM	Substandard and falsified medicines
UP	Universitas Pancasila
USD	American Dollar

B. Global Costing Reference Case principles applied to STARmeds cost analysis

**Reference Case Checklist Items**	**Options**
STUDY DESIGN AND SCOPE	
Principle 1 - The purpose of the study, the population, and the intervention and/or service/output being costed should be clearly defined.	
Purpose	
Purpose type:	Financial planning and budget Impact analysis.
Relevance for health practice and/or policy decisions:	Inform on the key aspects of sampling and quality testing medicines from the market, its objectives and resources.
Aim of the cost analysis:	Provide an estimate of costs of sampling and quality testing medicnes, based on STARmeds project

	implemented in Indonesia to study the prevalence of substandard and falsified medicines.
Intended user(s) of the cost estimate:	Medicine regulators or other researchers implementing medicine quality studies in the field.
Intervention	
Main activities/technologies involved:	Medicine quality study including sampling collection from the market and medicines quality testing.
Target population:	In the context of this intervention, the population can be considered as the medicines being collected in the field. In this case, the target medicines were allopurinol, amlodipine, amoxicillin, cefixime and dexamethasone collected from pharmacies, hospitals, health care professionals and online sellers.
Coverage level:	Sampling locations (districts) were chosen purposively to reflect Indonesia's geographic and economic diversity. Considering medicines as the target population, it is not possible to measure coverage level since it is extremely difficult to estimate the total number of medicines being sold physically or online in the country.
Delivery mechanism (e.g., health system level, facility type, ownership, etc.):	Medicines were sampled from pharmacies physical and online, hospitals, clinics and health care providers.
Epidemiological context (i.e., incidence/prevalence of disease)	Prevalence of substandard and falsified medicines in Indonesia is unknown. According to WHO in 2017, "1 in 10 medical products in developing countries is substandard or falsified".(5)
Intervention	Analysis and quality testing of medicines sampled from a random selection of outlets where patients obtain medicines. Sample collection was conducted with "mystery shopping" method for buying medicines from pharmacies and "overt sampling" for buying medicines from hospitals, clinics and health professionals. Field activities consisted on:<br>• Buying samples;<br>• Storing and delivering collected samples;<br>• Downloading and updating data entry forms;<br>• Filling and submitting forms.<br><br>After collection, samples were sent to lab testing. Samples that failed tests of quality were combined with information about the volume of medicines of each

	brand dispensed by different outlets to include different sources of risk that patients will be exposed to (see Supplementary file 3). Activities related to testing of medicines include:<br>• Visual inspection - packaging check<br>• Creating laboratory protocols - validation of testing protocols<br>• Send samples for lab testing.

Principle 2 - The perspective (extent of the resource use captured) of the cost estimation should be stated and justified relevant to purpose.	
Study perspective (e.g., provider, health system, societal, household):	Costing analysis was developed adopting the perspective of a medicines' regulator, excluding as much as possible, any costs associated with research.
Principle 3 - The type of cost being estimated should be clearly defined, in terms of economic vs financial, real world vs guideline, and incremental vs full cost, and whether the cost is 'net of future cost', should be justified relevant to purpose.	
Defining the cost	
Economic vs. financial cost	This costing study includes both financial and economic costs. Financial costs were extracted from the projects' administrative records and economic costs were computed from consequences of the projects' activities that were not included in the projects' expenses, such as overtime hours worked by staff or usage of personal equipment (phones, laptops, etc..).
Real world' vs guideline cost	We use real world data from administrative records that were continuously registered through the course of the whole project, from design to implementation of the medicine quality survey. We also include qualitative data from staff interviews and Indonesia civil service pay scale,

	qualitative data on workload from time-and-motion survey tools for measuring workload and overhead costs.
Full vs incremental cost	Full costing method.
Net of future cost	Not included.
Principle 4 - The 'units' in the unit costs for strategies, services and interventions should be defined, relevant for the costing purpose, and generalizable.	
List the unit costs used	• Unit costs for samples: cost per sample collected;<br>• Unit costs for outcome: cost per OOS found;<br>• Unit costs per time: Cost per week and per fieldwork day and week.
Describe any adjustments made to reflect the quality of service output	N.a.
Principle 5 - The time horizon should be of sufficient length to capture all costs relevant to the purpose, and consideration should be given to disaggregating costs into separate time periods where appropriate.	
Time period	
Period type (start-up vs implementation):	Start-up and implementation.
Time period:	October 2021 to October 2022

SERVICE AND RESOURCE USE MEASUREMENT
Principle 6 - The scope of the inputs to include in the cost estimation should be defined and justified relevant to purpose.
Defining the scope

Above service delivery costs included	Yes, medicines delivery costs to research hub and transportation to lab facilities were included.
Costs of supporting change included	No
Research costs included	No
Unrelated costs included	No
If incremental costs, assumptions made for existing capacity	N.a.
Any exclusions other to scope	N.a.
Principle 7 - The methods for estimating the quantity of inputs should be described, including data sources and criteria for allocating resources.	
Describe the measurement of each input as either top-down or bottom-up	We use a micro-costing model to estimate total costs of the sampling medicines from the market and testing for medicine quality, implemented by STARmeds project, with a top-down approach.
Describe method to allocate human resources inputs	Salary values were collected from the Indonesian civil servant salaries list, using the 2019 Government Regulation. To correspond the civil service positions and grade levels to the hypothetical team we created, we made a set of assumptions based on seniority. In addition, we use qualitative data from interviews with STARmeds staff to fairly account for overtime hours worked. These costs are computed by multiplying their full-time equivalent (FTE) salaries by the estimated number of hours worked outside normal working hours (assumed to be from 9am to 5pm).
Describe methods to allocate above site/overhead inputs	Overhead rates were attributed based on literature and consultation with local experts. It was assumed that overheads represent 10% of the sum of salaries, consumables, travel, and other direct costs.

Describe the methods for excluding research costs	All costs associated with research, for example studying sampling methods, were not including in the costing analysis. This is done mostly by including only costs incurred during medicines collection and testing, and by creating an hypothetical medicines regulator team that only includes members that would also be necessary by a regulator conducting the same type of activities (decided in consultation with local experts).
Describe the methods for measuring other resources	Equipment:<br><br>The cost of newly purchased equipment was informed by STARmeds expenditure records. When personal laptops and phones were used by the core team, purchase values of USD 1000 and USD 100, respectively, were used to compute economic costs, informed by prices of similar specifications to those used in STARmeds available on the e-catalog website of Indonesia's National Public Procurement Agency LKPP. In all cases, a useful life of 5 years and a discount rate of 3% were assumed to annualize equipment cost.<br><br>Consumables:<br><br>All costs of consumables were informed by STARmeds expenditure records. Covid-19 related consumables were excluded e.g., rapid diagnostic tests.<br><br>Travel This category includes travel, meals, accommodation, and meeting-related costs e.g., training data collectors. In each geographical location from where samples were collected, there was a central coordination hub for which rent, utilities, buildings insurance were charged at market prices. Transport between Jakarta and the provinces, as well as local transport, are also included. All costs are informed by STARmeds expenditure records.<br><br>Laboratory:<br><br>This category includes the costs of privately contracted laboratory tests for 1,274 medicine samples.* Informed by STARmeds expenditure records.<br><br>Other Direct Costs:<br><br>This category includes operational allowances for sample collectors, the cost of purchasing medicines from outlets at market prices, as well as a wide range of services and consumables e.g., barcoding samples, mobile data packages for the field team. All costs are informed by STARmeds expenditure records.
Principle 8 - The sampling strategy used should be determined by the precision demanded by the costing purpose and designed to minimize bias.	
Site/client selection process/criteria	

Describe geographic sampling (if applicable)	The STARmeds project was implemented in four medicines of Indonesia (North Sumatera, Jakarta, East Java and East Nusa Tenggara (NTT)). The choice of geographic area was part of a multi-stage random sample design.
Describe site sampling (if applicable)	N.a.
Describe patient sampling (if applicable)	N.a.
Describe methods to calculate sample size	Sample size was calculated based on the rule of thumb for single proportions, according to which a minimum of 138 samples would be needed to be 95% confident that a measured prevalence of 10% substandard or falsified medicines reflects the true proportion of poor-quality medicines.
Principle 9 - The selection of the data source(s) and methods for estimating service use should be described, and potential biases reported in the study limitations.	
Identify the data source used to measure the units	Data sources:<br>STARmeds financial and administrative records;<br>Civil servant's salaries lists (Government Regulation, 2019); and<br>Time-and-motion surveys with the expert staff in the field that provided crucial information on overtime worked by staff and overhead rates.
Where relevant describe the sampling frame, method and size:	The sampling frame for the cost analysis is the same as the sampling frame of the STARmeds project. STARmeds collected 1,333 samples, of which 1,274 were eligible for testing and 327 were collected online. STARmeds Guide and Toolkit includes a version of the study design description adaptable to many settings in chapter 3 (see Supplementary file 3) and the units of analysis for the costing study are defined based on information in STARmeds administrative records.
Describe any method used to fill missing data	No methods to fill in missing information were needed for the costing analysis.
Principle 10 - Consideration should be given to the timing of data collection to minimize recall bias and, where relevant, the impact of seasonality and other differences over time.	
The timing of data collection should be specified in the following ways:	
Timing of data collection	The time horizon covers the calendar period October 2021 – October 2022, during which the STARmeds study design, data collection and laboratory analysis took

(resource and service use)	place. The scope of the costing exercise includes all resources consumed by STARmeds related to all these stages of the study.
Prospective or retrospective	Retrospective study, referring to the period between October 2021 and October 2022.
Longitudinal vs cross-sectional data	We use longitudinal, daily and weekly data, collected through the development of the STARmeds project and cross-sectional data on workload reported by our staff members in a single point in time, about 6 months after data collection was concluded.
Recall period, where relevant	Workload and overtime were measured through interviews with STARmeds staff about 6 months after the conclusion of data collection.

**VALUATION AND PRICING**	
Principle 11 - The sources for price data should be listed by input, and clear delineation should be made between local and international price data sources, and tradeable, non-tradeable goods.	
Report the sources of price data by input	Source: Administrative records<br>List of inputs:<br>Planning and preparation:<br>• Stakeholder engagement<br>• Study design<br>• Operational preparations<br>• Recruit and train data collectors<br>Data collection:<br>• Fieldwork, physical outlets<br>• Online outlets<br>• Sample processing<br>• Progress monitoring<br>• Triage and shipping<br>Reporting and analysis:<br>• Write up data collection report<br>• Record and review results from lab<br><br>Source: Indonesian Civil Service salary according to the 2019 Government regulation<br>List of inputs:

	All study phases:<br>• Salary rates and professional level assumed for FTE calculations.<br><br>Source: Time-sheets and interviews<br>List of inputs:<br>All study phases:<br>• Overtime hours worked per stage;<br>• Tasks duration;<br><br>Source: Literature and consultation with experts<br>List of inputs:<br>All study phases:<br>• Overhead rate.
Report inputs where local and international prices were used	All costs are reported is United States Dollar (USD, current 2022 values). Since STARmeds expenditure reports were submitted in GBP to the study funder, these amounts were converted to USD based on the average Great British Pound (GBP) to USD exchange rate in 2022. Other costs not incurred in GBP were converted from Indonesian Rupiahs (IDR) to USD using the average IDR to USD exchange rate for 2022. Given the one-year horizon (October 2021 – October 2022), no discounting was applied.
Principle 12 - Capital costs should be appropriately annuitized or depreciated to reflect the expected life of capital inputs.	
Describe the depreciation approach	For equipment, we assume a useful life of 5 years and a discount rate of 3% to annualize costs.
Describe any discount rate used for capital goods	N.a.
Report the expected life years of capital goods, and data sources	An online dashboard based on this costing analysis will be available with open access for public consultation.
Principle 13 - Where relevant an appropriate discount rate, inflation and exchange rates should be used, and clearly stated.	

Describe any discount rate used for future costs	No discounting was applied.
Describe the reported currency year	All costs are reported is United States Dollar (USD, current 2022 values).
Describe any conversions made	Expenditures submitted in GBP were converted to USD based on the average Great British Pound (GBP) to USD exchange rate in 2022.<br><br>Other costs were converted from Indonesian Rupiahs (IDR) to USD using the average IDR to USD exchange rate for 2022.
Report the inflation type and rate used	N.a.
Principle 14 - The use and source of shadow prices for goods and for the opportunity cost of time should be reported.	
Methods for valuing the following should be reported:	
Report methods for valuing volunteer time	We record overtime hours worked by STARmeds staff through interviews with those who were working in the field. Of all the project stages, data collection was the most intense and required staff to be available to work past the normal working hours for about two months.
Report adjustments for input prices (donated or subsidized goods)	N.a.
**ANALYZING AND PRESENTING RESULTS**	
Principle 15 - Variation in the cost of the intervention by site size/ organization, sub-populations, or by other drivers of heterogeneity should be explored and reported.	
Describe any sub-groups or populations analyzed	Cost analysis outputs consist in cost per day and per week of fieldwork, per sample collected and per out-of-specification sample (OOS). These average costs were calculated using bootstrapping method to capture uncertainty in the number of samples collected, by seller location type (urban, rural, and online).
Describe any statistical methods used to establish differences in unit costs by sub-group	Differences in unit costs by sub-groups were not applicable.
Describe any determinants of cost (model specification)	In this micro costing study we aggregate costs by study phase and item, studying the relative weight of each component on the total costs.

Describe any multivariate statistical methods used to analyze cost functions	No multivariate statistical methods were applied.
Principle 16 - The uncertainty associated with cost estimates should be appropriately characterized.	
Describe sensitivity analyses conducted	We include a one-way sensitivity analysis, with three alternative scenarios where we change average values found using STARmeds (keeping all other costs constant):<br>A) Increase costs of sampled medicines,<br>B) Decrease preparations costs, and<br>C) Increase OOS prevalence estimates
List possible sources of bias	Although we did not include research costs, STARmeds project consists in a cross-section survey of medicines at poin of dispensing, designed and conducted by academics, which can result in a bias towards more academic methods. Medicines collected by STARmeds were considerably cheap relative to the market average, which can result in an underestimation of costs.
Principle 17 - Cost estimates should be communicated clearly and transparently to enable decision-maker(s) to interpret and use the results.	
Limitations	
Limitations in the design, analysis, and results	Difficult to completely capture how a medicines' regulator would implement the same type of medicine quality study using an example from a research team.<br>We did not factor in the cost of policy action towards OOS samples (e.g., inquiries, checks, inspections, other administrative or legal action) or the potential deterrent effect of such action.<br>Potential recall bias and data inconsistencies when estimating the workload amount.
Aspects of the cost estimates that would limit generalizability of results to other constituencies	STARmeds project collected medicines only on 5 medicines that are some of the cheapest medicines in the Indonesian pharmaceutical market. This can affect the generalization of the results to medicines quality studies conducted for other types of medicines and other countries.
Conflicts of Interest	

All pecuniary and non-pecuniary interests of the study contributors	N.a.
All sources of funding that supported conduct of the costing	The STARmeds study is funded by the UK National Institute for Health Research through the Global Health Policy and Systems Research Program, grant number NIHR131145.
Non-monetary sources of support for conduct of the costing	N.a.
Open access	
Dataset available	Dataset is available upon request.
