# Supplementary material for: Value for money of medicine sampling and quality testing: evidence from Indonesia
Source: BMJ Glob Health. 2024 Sep 23;9(9):e015402. doi: 10.1136/bmjgh-2024-015402 (PMC11429347; doi:10.1136/bmjgh-2024-015402)
Supplement: online supplemental file 2 [file bmjgh-9-9-s002.pdf]

Supplementary file 2. Additional tables

Table A2.1 Medicines tested per molecule

Molecule name	Quality test outcome		Total
	Pass	Fail	
Allopurinol	279	18	**297**
Amlodipine	240	4	**244**
Amoxicillin	269	30	**299**
Cefixime	158	37	**195**
Dexamethasone	223	16	**239**
**Total**	**1,169**	**105**	**1,274**

Source: STARmeds prevalence estimates (14).

Supplementary file 3. STARmeds Guide and Toolkit Chapter 3 (generalizable version of the STARmeds study design)

Conducting Randomised Field Surveys of Medicine Quality Using Mystery Shoppers: A Practical Guide and Toolkit (13)

Study design

Final study design requires detailed decisions about sampling locations and levels, as well as about which products to sample, and how many samples should be collected. These will all flow from the overall purpose of the study, and available resources.

Study aim

Studies that collect and test medicines from a random sample of outlets at which patients access those products generally aim to measure the proportion of samples that do not meet quality standards; or the proportion of outlets that dispense or sell medicines that do not meet quality standards; or both. Within that broad goal, a study may also be designed to compare measures of quality across a number of different dimensions, for example:

- Geographic area of sale
- Therapeutic class
- Price point or brand status
- Type of outlet or source
- Country or area of production

If the intention is to compare prevalence on any of these dimensions, then it is necessary to ensure that sample sizes for each group (for example for each province, or for domestically produced and imported medicines) is large enough to allow for a statistically significant difference to be measured (see section on sample size calculation below).

Choosing medicines

Medicine quality testing involves the use of a (usually expensive) reference standard, and a validated testing protocol, the latter specific to the formulation of a medicine (for example, it differs for tablets, capsules or dry syrup formulations of the same molecule). This usually limits the number of medicines and formats that can be sampled in any study. In practice, the choice of medicines is often dictated by the institutional interests of the providers of research funding. However, in order to maximise utility to countries where research takes place, other factors should also be considered when choosing medicines.

Public health importance

Medicines may be chosen for study based on the magnitude of the risk to public health if that particular medicine were found to be substandard or falsified. Measuring public health importance is not straightforward. It can include measures of:

- Disease prevalence: how many people are affected by the condition the medicine treats?
- Patient vulnerability: does the condition affect especially vulnerable people, such as young children, or people most likely to lack health insurance or access to public services?
- Medical consequences of failure: how severely would a patient's health be affected if the medicines were substandard? For example, the consequences of substandard paracetamol may be a prolonged headache, while substandard chemotherapy may lead to death. Medicines with a "narrow therapeutic index" -- including those that are toxic if over-dosed -- may have especially severe consequences.
- Secondary effects of poor quality: for example, substandard antimicrobials could contribute to antimicrobial resistance, so that even quality medicines become ineffective.
- Budgetary impact: does the medicine represent a high burden on the national or on family budgets?

Quality risk

Some medicines are at higher risk for being substandard or falsified than others, either for reasons inherent in the product, or because of market factors. Studies may wish to focus on medicines at higher risk, though if this choice is made, it is important not to imply that the prevalence of poor quality in these medicines represents all medicines on the national market.

Potential indicators of increased risk for substandard products include:

- Chemical composition: some medicines are less stable than others; less-stable medicines and those which are temperature-sensitive are especially likely to degrade;
- Formulation: complex production techniques or sterile formulations may be at particular risk for production errors;

- Variety of producers: the more producers there are of a locally-marketed molecule (including producers in other countries), the more likely it is that some producers will fail to meet quality standards;

Potential indicators of increased risk for falsified products include:

- Medicines with severely controlled or restricted distribution: restrictions may apply because medicines are used recreationally, or for purposes not authorised locally. Narcotics, psychotropics, steroids and abortifacients often fall into this category;
- Medicines that are very expensive, and not covered by health insurance

Feasibility

The choice of medicines have important implications for feasibility of sampling. Before finalising the choice of study medicines, the following issues should be considered:

- Affordability: can the medicine be bought and tested within the available budget?
- Accessibility: can the medicine be easily acquired from the outlets included in the study design, in the volumes necessary for testing? This consideration often rules out the inclusion of controlled medicines such as narcotics, and in some settings makes it difficult to sample some antibiotics;
- Handling needs: it may not be practical to include medicines that must be stored and transported in particular ways, for example cold-chain products;
- Capacity for testing: is there a laboratory able and willing to test the medicines and formulations chosen?
- Political acceptability: global health organisations, research funders and governments may have programme priorities that do not reflect local public health needs very closely. It will usually be easier to get support for the investigation of medicines that treat or prevent conditions that are current political priorities, as well as to engage potential users with the results of such studies, than it will be to study medicines relating to health areas that are not currently in fashion.

To the extent possible, all of these decisions should be based on recent data. In terms of public health importance, for example, data about procurement volumes and values can provide a crude ranking of the importance of a medicine in the public system. Bear in mind, however, that raw volumes do not translate easily into disease prevalence or patient exposure, because pills per patient vary, and medicines for chronic conditions may be taken indefinitely, while those for acute conditions will be taken only for a short period. The consultations described in Step 2 on page 8 are essential in order to access reliable, recent data, as well as to canvas opinions about which factors to prioritise in choosing study medicines.

Choosing study areas

The choice of geographic areas in which to collect samples was influenced by the overall purpose of the study. Choice of geographic area may be part of a multi-stage random sample design (see section

on randomisation techniques on page 18). However, this is likely to be expensive, and feasibility may be low. The alternative is to choose sampling areas purposively.

Explanatory potential

The areas included in the study have an important bearing on the interpretation of the results. These include:

- Geographic diversity: even if a study is not designed specifically to compare prevalence rates between areas, researchers may wish to ensure that different regions are included, based on population density, economic development, ethnic or other demographic profiles, or other factors;
- Political or administrative diversity: in countries with decentralised health systems, it may be useful to include areas with different models of medicine procurement or dispensing.
- Comparative data: some areas may be richer than others in information which would add to the learning generated from the study (for example previous studies of medicine quality, or of medicine procurement or dispensing).

Supply chain and quality risks

In some country settings, falsified (and in rarer cases substandard) medicines may cluster geographically. For example, unregistered and other smuggled medicines might be found in areas bordering countries with weaker medicine regulation or greater availability of affordable medicines. Similarly, informal markets -- more easily penetrated by falsifiers -- might be concentrated in a particular district or along a trade route. Studies may wish to include or focus on these higher risk areas. If this choice is made, it is important not to imply that the prevalence of poor quality in these areas represents all medicines on the national market.

Feasibility

Issues to consider include:

- Density of outlets and stock levels: in some areas, for example remote rural districts, it may be difficult to buy enough pills to meet testing needs, or to sample from a large enough variety of outlets;
- Local support: the willingness of local authorities or research partners to support or allow the research may increase the likelihood that study results contribute to useful policy changes if needed.

Data that may help inform the choice of districts includes data on population density and per capita measures of health service availability and use, together with regulatory data on the historical distribution of detected cases of falsified or substandard medicines.

Choosing outlet types

The country context and the study design will influence decisions about which types of facilities data collectors will get samples from.

Representativeness

As with choice of geographic area, the included outlet types will influence the extent to which any measured prevalence can be generalised to a nation or region. Things to think about include:

- Inclusiveness: do the outlets chosen represent all the sources from which patients acquire medicines in the study area? Many national authorities restrict post-market surveillance to the regulated supply chain which they oversee. However, in many countries, patients also buy medicines from sources not technically authorised to sell them, such as street vendors, internet vendors, or private midwives, nurses or doctors. All of these de facto sources of medicines should be considered when choosing outlets for sampling.
- Patient exposure: do the outlets chosen represent the sources from which patients get most of their medicines, by volume? In some settings, a small number of outlets account for a significant proportion of dispensed medicines. (For a detailed description of exposure-based sampling see (29))

Quality risks

In most countries, falsified products are much more commonly found in the unregulated supply chain (including unlicensed internet vendors and informal markets) than in regular outlets such as hospitals or pharmacies which are subject to rules and oversight. Studies may wish to include or focus on these higher risk outlets. If this choice is made, it is important not to imply that the prevalence of poor quality in these outlets represents all medicines on the national market.

Feasibility

Not all outlet types are equally accessible to sampling staff. Issues to consider include:

- Availability of target medicines: some medicines are only available in hospital settings, or other service-specific settings. In addition, some types of outlets do not maintain significant stocks of medicines, so it may be difficult to acquire enough pills, bottles or vials to make up a viable sample.
- Permissions and ethical challenges: sampling from some outlets, often including hospitals and health centres, may require additional permissions; "mystery shopper" sample collection techniques may not be possible so overt sampling may be needed. In areas with limited stocks, there is a risk that taking samples for the study might deprive patients of medicines they need -- a situation that must be avoided for ethical reasons.
- Data sources for sample frame: random sampling presupposes that each outlet has an equal chance of being selected; a full listing or mapping of potential outlets is thus 13 needed. Some outlets, such as itinerant medicine vendors, may be especially difficult to include in a random design if resources for mapping are not available.

Sample size calculations

The number of samples required depends on the questions researchers want to answer. If the study aims to make statistically valid comparisons between the prevalence of poor quality among medicines with different characteristics (for example comparing the quality of antibiotics with that of

cardiovascular medicines, or comparing medicine quality in rural areas with that in urban areas), each comparison must be taken into account when calculating the minimum sample size. Free, on-line tools that calculate the sample size needed to estimate a single proportion (prevalence) or the difference between proportions are available from several sources. Examples include https://statulator.com/ and the Statcalc function in EpiInfo (https://www.cdc.gov/epiinfo/support/downloads.html). These require users to input the desired levels of accuracy and an estimate of the baseline prevalence of poor-quality drugs, or to determine the difference between proportions. As a general rule of thumb, and following the equations for single proportions described in the sources above, 138 samples would be needed to be 95% confident that a measured prevalence of 10% substandard or falsified medicines reflects the true proportion of poor-quality medicines. Calculating sample sizes to estimate the difference between two proportions accurately requires researchers to decide what level of difference is worth measuring. For example, if we wanted to be able to measure a 50% difference in prevalence between rural and urban areas -- assuming for example that 14% of medicines in rural areas are substandard compared with 8% of medicines in urban areas, we would need to sample 929 medicines in each area to be 95% confident that our estimates reflect the true difference. The number of units (pills, bottles or vials) required for each sample is discussed under testing parameters, in the next step.

Supplementary file 4. Civil Service salary levels calculations

We create a hypothetical team from a regulatory agency working on medicine quality surveillance to estimate potential salaries expenses from a regulator's perspective. Salary values were collected from the Indonesian civil servant salaries list, using the 2019 Government Regulation. To correspond the civil service positions and grade levels to the hypothetical team we created, we made a set of assumptions based on seniority. There are 4 grades of civil servant in Indonesia, with grade I being the lowest and IV the highest. Generally, each grade corresponds to the following types of professions:

- Grade I: Employee
- Grade II: Admin
- Grade III: Coordinator/Manager
- Grade IV: Supervisor

In our hypothetical team, we correspond grade I to assistant level, grade II to data manager, grade III to project manager and grade IV to principal investigator (PI).

The salary values publicly available in the Government Regulation include only basic salaries. In addition to that, civil servants receive allowances from the local government, depending on their position and the province they work at. There are several types of allowances, including for performance, spouses, children, position, workload, transportation, meal, and work traveling, that can go up to 4 or 6 times the basic salary. To make a realistic assumption of these values for our hypothetical team, we consider allowances for the PI are four times their basic salary, for the project manager allowance is the triple of the basic salary, for data manger the double, and for an employee the allowance is the same value as the basic salary.

Salaries and allowances were computed per worker per month and converted from IDR to USD currency at 2022 values. Table A4.1 summarizes the assumptions described above.

Table A4.1 – Monthly salary assumptions (in USD, 2022 values)

Hypothetical team	Assumed Indonesian CS position	Basic salary	Allowance	Total salary
Principal Investigator	Grade IV (a-e): Supervisor	USD 223.1	USD 892.3	USD 1115.4
Project Manager	Grade III (a-d): Coordinator	USD 185.2	USD 555.5	USD 740.7
Data Manager	Grade II (a-d): Admin	USD 147.5	USD 294.9	USD 442.4
Assistant	Grade I (a-d): Employee	USD 107.0	USD 107.0	USD 213.9

Source: 2019 Government Regulation and own assumptions.(16)

Applying these salary rates to the hypothetical core medicine quality team that we assume for calculating HR costs in our analysis, the resulting salaries for each position and percentage of time spent on STARmeds are presented in table A4.2.

Table A4.2 Salaries tables – Hypothetical team

Role	Annual salary USD	% of working time spent on STARmeds, by phase		
		Preparation	Data collection	Analysis and reporting
Principal Investigator	10217	50%	50%	50%
Project Manager	6785	100%	100%	100%
Data Manager	4052	100%	100%	100%
Assistant 1	1960	80%	80%	80%
Assistant 2	1960	80%	80%	80%
Assistant 3	1960	80%	80%	80%
Assistant 4	1960	80%	80%	80%

Source: 2019 Government Regulation and own calculations. (16)

Supplementary file 5. Time-and-motion study

Methods

The STARmeds data collection can be divided into two major groups of operations, sample management and field work (Figure A5.1 Activity-based Costing Diagram). Sample management was led by the research team and corresponds to all the activities that made the field work successful.

These were divided in three main stages of preparation and planning, data collection support and stakeholder engagement, ongoing between September 2021 and May 2022. Field work corresponds to all the activities directly involved in samples collection, either physically or online, from going to the pharmacy or registering an account with an online seller. The field work was executed throughout three months, between mid-February to mid-May 2022.

Sample management

The STARmeds research team designed and provided support to all data collection related activities, ensuring its flow and success in achieving the target number of samples within a limited timeframe (Figure A5.2 shows STARmeds perations flow-chart). To understand the use of time and workload involved in this process, a series of three focus group discussions were conducted, after which each team member completed a time-use individual questionnaire on the specific activities they were involved in. Focus groups discussions happened around six months after data collection was finalized and were organized by phase of the project. The first discussion covered activities performed during planning and preparation, the second focused data collection support in the field and the last was on engagement with stakeholders. Each focus group lasted about one hour, and the invitation was sent to all staff members. Each meeting was led by the STARmeds economic evaluation team, using guidelines for discussion that were outlined together with team members directly involved in data collection. The main objective of these focus groups was to create safe environments for staff to share their experience in the field and how they organised their time during that period. Focus groups were also useful to clarify key concepts and text interpretations relevant to complete the questionnaire.

After each focus group, staff completed an anonymous questionnaire that asked them to indicate their main role in STARmeds, in which phase of preparation, data collection and stakeholder engagement they participated on, how they allocated their time between these phases, how many hours they worked per week in each activity of each phase, how many days a week they worked overtime for each activity and in what months they worked on each activity the most. They were also asked to rank their activities from most to least time-consuming within each phase.

To translate these data into costs, overtime was counted at the same rate as standard working hours (based on Indonesian civil service salary rate) and the adjustment was made in terms of duration of the project, i.e., how many extra days would have been needed for researchers to complete the same amount of work without compromising their free time. Salaries were adjusted to the time allocation reports to account for the fact that data collection preparation and execution activities only occupied a fraction of the researcher's time. For example, if data collection related activities occupied only 20% of a data analyst time, to get the staff costs associated to data collection only, we need to multiply the salary by 20%, the percentage of the salary that was actually spent on data collection.

Data collectors training was provided on-line and in person by STARmeds research staff. Two members of the research staff were actively involved in recruitment and training of data collectors for about 1 to 2 hours per day, for two weeks. Once in the field, data collectors often needed to confirm aspects related with prescriptions, missing medicines, stock shortages, that needed a quick and clear response from the research staff. This support was mostly done through back and forward messaging using WhatsApp social network.

Engagement with stakeholders happened throughout the whole period of preparing and executing data collection and was crucial to ensure the collaboration of local authorities and partner institutions. By stakeholders we mean every person or institution that somehow had an impact in our sampling activities, from the highest level and the Ministry of Health to the district public health facility doctor. The need and process of acquiring administrative permissions was very different across sites and type of medicine. In general, to get high-level permits, the first step was getting in contact with high level stakeholders from public health departments, which could take from three weeks to several months. From there, the permit took from 2 to 3 weeks, depending on the required documents (e.g., recommendations letters). In other regions, the permit application process was not fully formalized, and it implied a lot of back and forward between different levels of institutions and hospitals. At the same time, for some locations there were online platforms to apply for data collection permits that were quite straightforward and effective.

Field work

To collect time use data of field activities, enumerators were requested to register timestamps systematically through the execution of their tasks. To ensure the consistency and quality of these data, data collectors training included sessions on how to properly fill the electronic and paper forms. All sample collectors were requested to register each sample electronically using KoboToolBox software. The data entry form included automatically registered timestamps to measure the time each enumerator took to register the sample collected. Data collectors conducting mystery shopping not only recorded their timestamps when registering the samples electronically, but also filled in paper forms prepared by the research team.

Online buying was executed by 23 data collectors, of which 10 were STARmeds research staff who collected most of the samples (73%). The electronic form monitored the execution of 4 different tasks when buying each sample online: open the form, start shopping, finalized for and closing the form.

Results on average time between tasks by type of area (rural, urban or online) are presented in Table A5.1. Figure A5.3 shows average time from getting to the store to filling the form in the different regions where data was collected.

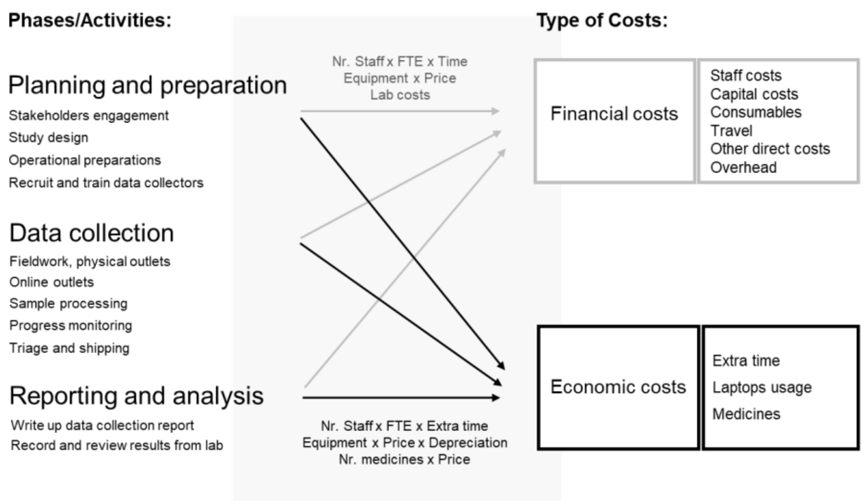

Figure A5.1 Activity-based Costing Diagram

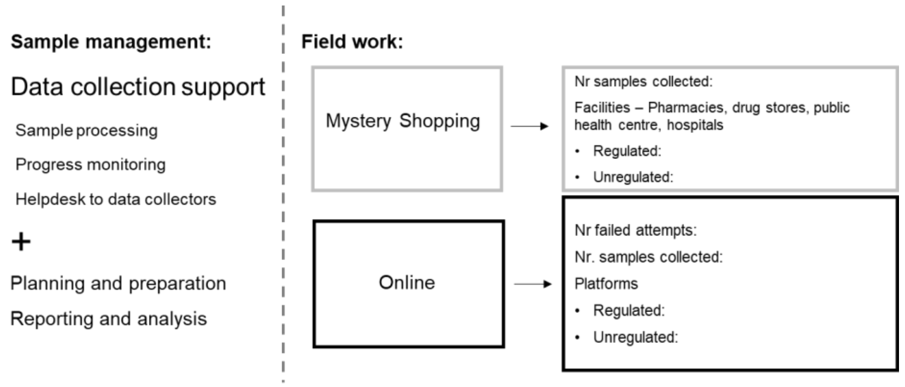

Figure A5.2 Operations flow-chart

Table A5.1 – Data collected mystery shopping and online

**Area**	**Number of Enum**	**Number samples (STATA)**	**Samples per enumerator**	**Days in field**	**Samples per day**	**Time between tasks (min.)***
**Urban**	45	775	17,2	30	25,8	18,425
**Rural**	16	231	14,4	14	16,5	23,3
**Online**	45	329	7,3	46	7,2	

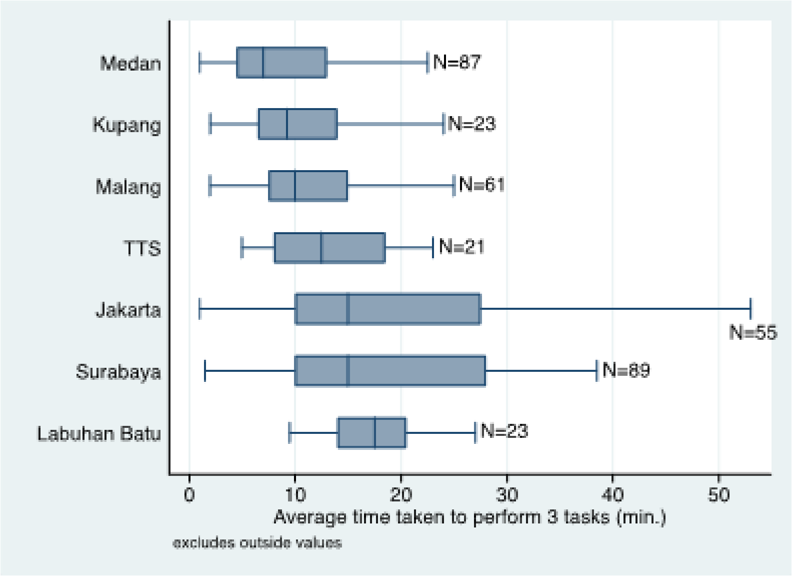

Figure A5.3 - Data Collection average time from getting to the store to filling the form
